# The mevalonate coordinates energy input and cell proliferation

**DOI:** 10.1038/s41419-019-1544-y

**Published:** 2019-04-11

**Authors:** Li Gong, Yi Xiao, Fan Xia, Pei Wu, Tingting Zhao, Shulin Xie, Ran Wang, Qiaocheng Wen, Wensu Zhou, Huilan Xu, Lingyan Zhu, Zeqi Zheng, Tianlun Yang, Zihua Chen, Qiong Duan

**Affiliations:** 1 Department of Cardiology, Xiangya Hospital, Central South University, 87 Xiangya Road, Changsha, China; 2 Department of Cardiology, The First Affiliated Hospital of Nanchang University, Nanchang, China; 3 Jiangxi Hypertension Research Institute, Nanchang, China; 4 Department of Anesthesiology, Taojiang People’s Hospital, Taojiang, Hunan China; 5 Department of General Surgery, Xiangya Hospital, Central South University, Changsha, China; 6 Department of Social Medicine and Health Administration, Xiangya School of Public Health, Central South University, Changsha, China; 7 Department of Endocrinology, The First Affiliated Hospital of Nanchang University, Nanchang, China

## Abstract

The mevalonate pathway is known for the synthesis of cholesterol, but recent studies have reported that it also controls Hippo signaling, which is critical for the regulation of organ size and tumorigenesis. Here, we discover that the suppression of the mevalonate pathway inhibits the growth and proliferation of colon cancer cell lines. The results of transcriptomic and proteomic assays suggested that the mevalonate pathway controls multiple signaling pathways relevant to cell proliferation, and the results were further confirmed using western blot, PCR, and immunofluorescence assays. As cell proliferation is an energy-consuming process, we postulate that the mevalonate pathway may also control nutrient uptake to coordinate the processes of energy supply and cell proliferation. Here, we found that lovastatin, a mevalonate pathway inhibitor, suppresses glucose and amino acid uptake and lactate acid production. More importantly, mevalonic acid itself is sufficient to promote glucose uptake by colon cancer cells. In addition, we found that colon cancer tissues displayed a higher expression of mevalonate pathway enzymes, which may promote cell growth and stimulate energy uptake. Together, our findings establish the mevalonate pathway as a critical regulator in coordinating energy input and cell proliferation.

## Introduction

Cell proliferation and growth are under the tight control of intracellular signaling pathways and the extracellular environment, such as energy availability. How cells sense the extracellular nutrients and utilize them for growth and proliferation has been extensively studied^1–5^. Amino acids are the classical stimulus for mTORC1 activation^6,7^. In the presence of amino acids, mTOR promotes growth by stimulating the de novo synthesis of proteins, nucleotides, and lipids, and by inhibiting autophagy through the phosphorylation of ULK1 at Serine 758^4,8–10^. Glucose is the major source of energy for the cell. Recent studies showed that the Hippo pathway and AMP-activated protein kinase (AMPK) were activated during glucose starvation. Cellular energy stress, e.g. glucose withdrawal, induces YAP phosphorylation and cytoplasmic localization, as well as proteasomal degradation^2,3,11^. As a key transcription factor that induces cell growth and proliferation, YAP is also regulated by the condition of cellular energy supply.

The mevalonate pathway is known to synthesize cholesterol. HMG-CoA synthase and HMG-CoA reductase are rate-limiting enzymes catalyzing the conversion of acetyl-CoA to mevalonic acid (MVA)^12^. HMG-CoA reductase is the target of statins, which are commonly used for lipid-lowering therapy in patients with high-cholesterol. Statins have been shown to suppress the proliferation of cancer cells^13–15^. In addition, some studies have shown that statin use slightly decreased the risk of certain types of cancer, such as colon cancer^16–18^. Interestingly, epidemiological data also showed that statin use increased the risk of diabetes^19–22^. The evidences suggest that the mevalonate pathway is involved in the regulation of cell proliferation^23–25^, and probably, to control energy homeostasis simultaneously.

Two independent studies reported that statins could significantly suppress the nuclear localization and transcriptional responses of YAP and TAZ, two transcription factors that are influenced by energy supply^2,3^. Based on these findings, we postulate that the mevalonate pathway may function as a mediator to coordinate nutrient uptake and cell proliferation. In this study, we revealed that MVA, a key intermediate product of the mevalonate pathway, is essential for cell growth and proliferation. Transcriptome and proteome sequencing analysis showed that MVA activated multiple pathways responsible for cell growth and proliferation. MVA also promoted glucose and amino acid uptake, which orchestrates the cell proliferation. In addition, compared to the normal colon tissue, the colon carcinoma has increased the mevalonate pathway activity, represented by a higher expression of HMGCR and HMGCS1. These data establish the mevalonate pathway as a mediator that coordinates cell proliferation and nutrient uptake.

## Results

### The mevalonate pathway controls cell growth and proliferation

**Fig. 1:**
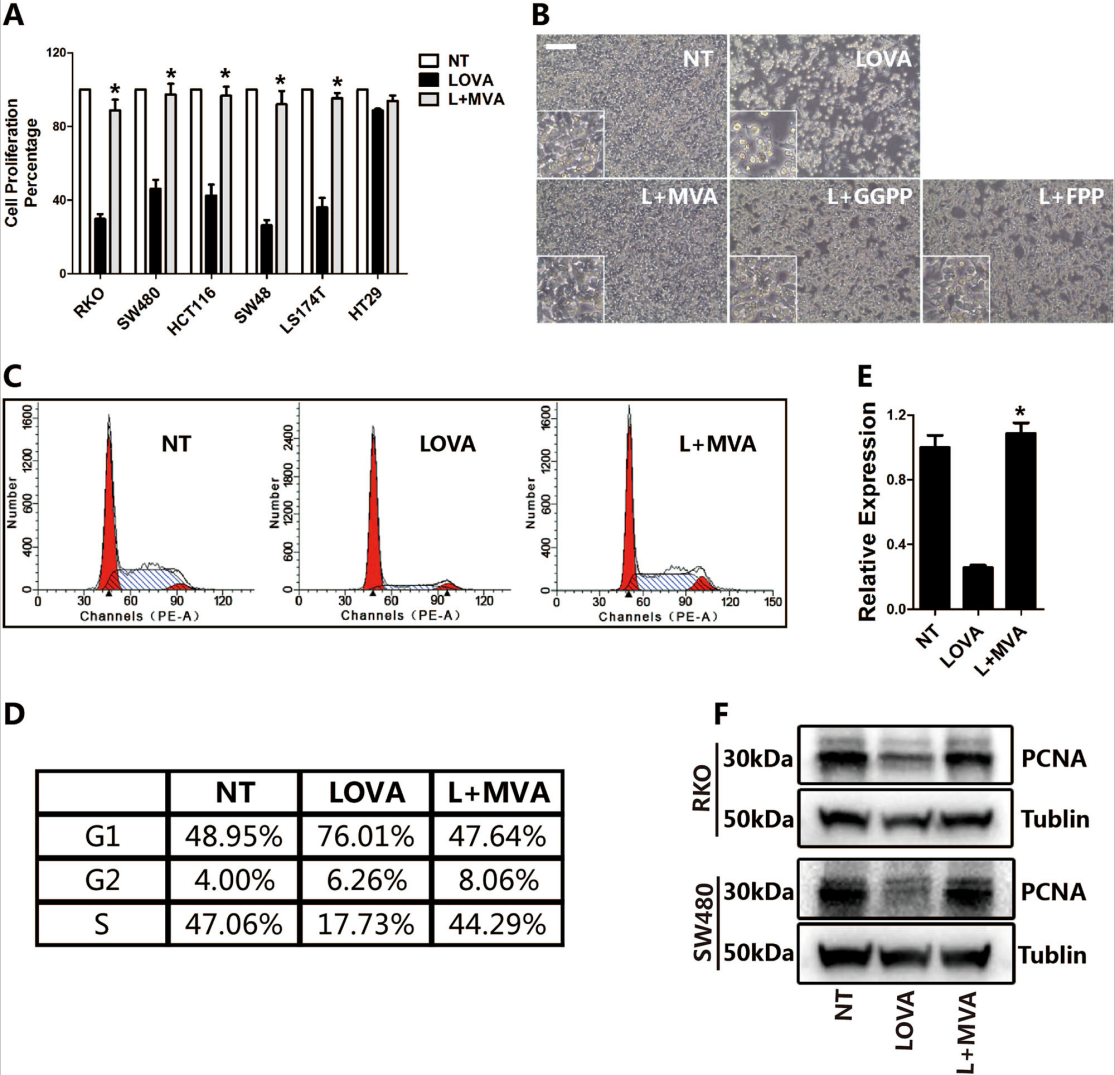
The mevalonate pathway controls cell growth and proliferation. **a**, **b** Lovastatin treatment suppresses the proliferation of colon cancer cells, and exogenous MVA restores the cell proliferation. The cells are treated with lovastatin (5 μM) or lovastatin plus MVA (0.5 mM) for 48 h, then the ATP level is measured to evaluate the cell proliferation (**a**) or take pictures under the optical microscope to observe the cell morphology (**b**). **c**, **d** Lovastatin treatment induces cell cycle arrest, but exogenous MVA releases the arrested cell cycle. RKO cells are treated with lovastatin or lovastatin plus MVA for 48 h and followed by cell cycle analysis using flow cytometry. **e**, **f** Lovastatin treatment downregulates the RNA (**e**) and protein expression (**f**) of PCNA, but exogenous MVA recovers the expression to a level of no-treatment condition. RKO or SW480 cells are treated with lovastatin or lovastatin plus MVA for 48 h, then the RNA and protein are isolated for RT-PCR or western blot assays. Data are expressed as means ± SEM. *P < 0.05 (LOVA versus L + MVA)

**Fig. 2:**
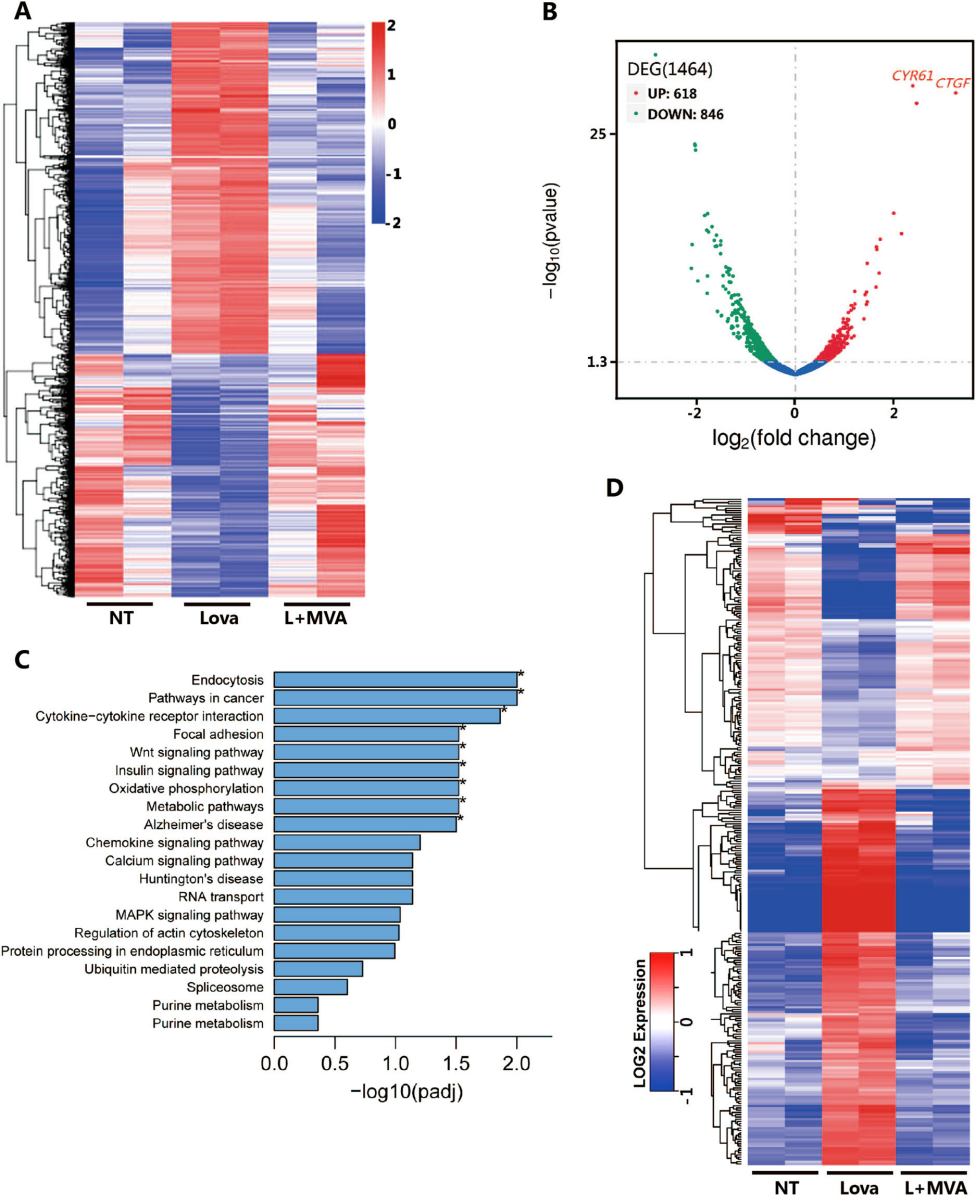
Omics studies of RKO cells treated with lovastatin or lovastatin plus MVA. **a** Heatmap depicting the differentially expressed genes detected by the RNA sequencing assay in RKO cells. RKO cells are treated with lovastatin (5 μM) or lovastatin plus MVA (0.5 mM) for 48 h and total RNA is isolated for RNA sequencing. **b** Volcano plot depicting the differentially expressed genes detected by the RNA sequencing assay in RKO cells (lovastatin plus MVA versus lovastatin). CTGF and CYR61 are among the most upregulated genes with MVA addition. **c** KEGG enrichment of the differentially expressed genes of the RKO cells. **d** Heatmap depicting the differentially expressed proteins detected by the proteomic assay in RKO cells. RKO cells were labeled as described in Method. Once labeled with isotopic amino acids, RKO cells are treated with lovastatin (5 μM) or lovastatin plus MVA (0.5 mM) for 48 h and then proceeded for the proteomic assay

Wnt and YAP/TAZ are two signaling pathways that control cell growth and proliferation. Given their key roles in the pathogenesis of colon cancer, we used colon cancer cell lines to test the effect of lovastatin on cell proliferation. The results show that proliferation was significantly suppressed by lovastatin treatment in all cell lines tested, except for HT-29 (Fig. 1a and S-Fig. 1A–D). The cell number and morphology were completely recovered by the addition of exogenous MVA (Fig. 1a, b). Geranylgeranyl pyrophosphate (GGPP) and farnesyl pyrophosphate (FPP), two intermediate products downstream of mevalonate, also recovered proliferation to some extent, but not as effectively as MVA (Fig. 1b and S-Fig. 2A). Cell cycle analysis performed by FACS confirmed that lovastatin increased cell G0/G1 arrest, while the addition of MVA released the arrest (Fig. 1c, d). Proliferating cell nuclear antigen (PCNA) is a marker of DNA replication and cell division. We therefore tested PCNA expression at both the RNA and protein level. The results showed that lovastatin treatment significantly downregulated RNA and protein expression in RKO and SW480 cells, and the addition of exogenous MVA recovered the expression (Fig.1e, f and S-Fig. 2B).

### Omics studies to explore the mechanism by which lovastatin suppresses proliferation

**Fig. 3:**
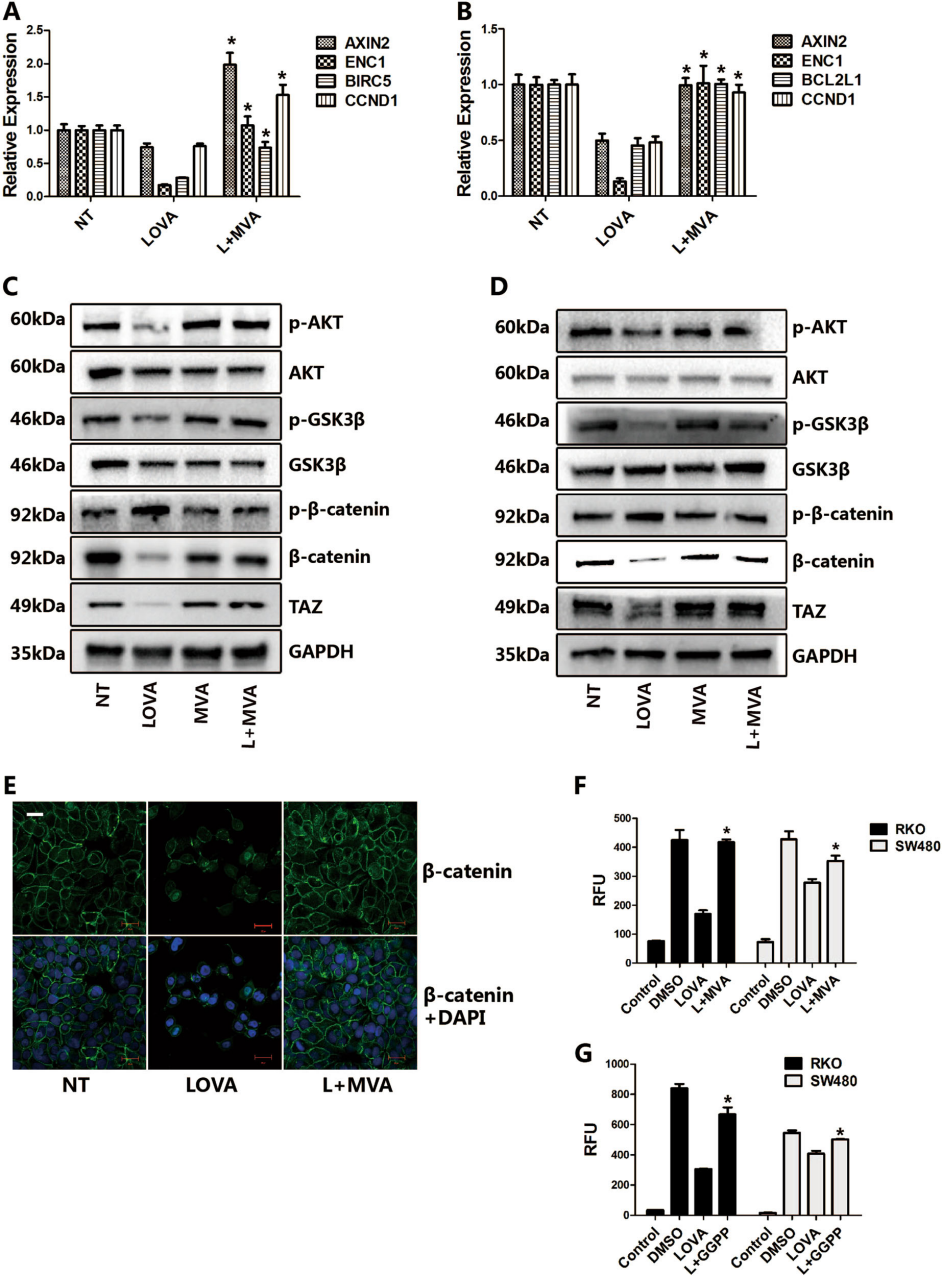
The mevalonate pathway regulates canonical Wnt signaling. **a**, **b** Lovastatin treatment suppresses the expression of Wnt target genes, while exogenous MVA restores them. RKO (**a**) or SW480 (**b**) cells are treated with lovastatin (5 μM) or lovastatin plus MVA (0.5 mM) for 48 h, and RNA is isolated for the RT-PCR assay. **c**, **d** Western blot assays of RKO (**c**) and SW480 (**d**) cells treated with lovastatin or lovastatin plus MVA. RKO or SW480 cells are treated with lovastatin (5 μM) or lovastatin plus MVA (0.5 mM) for 48 h, then the protein is isolated for the western blot assay. **e** Lovastatin treatment decreased the protein level of β-catenin in SW480 cells. Cells are treated with lovastatin (5 μM) or lovastatin plus MVA (0.5 mM) for 48 h, then immunofluorescence is performed to detect the β-catenin distribution. **f**, **g** Lovastatin treatment suppresses the activity of TCF/LEF reporter, while MVA (**f**) or GGPP (**g**) recovered it. TCF/LEF luciferase reporter is transfected to RKO or SW480 cells, then the cells are treated with lovastatin (5 μM) or lovastatin plus MVA (0.5 mM) for 48 h, and then the luminescence is measured. Data are expressed as means ± SEM. *P < 0.05 (LOVA versus L + MVA or L + GGPP)

To explore the underlying mechanisms by which lovastatin suppresses cell proliferation, we performed RNA sequencing and proteomics analysis. RNA sequencing results demonstrated that lovastatin treatment considerably changed the transcriptional profile of RKO cells (Fig. 2a, b and S-Tables 1–3). Compared to lovastatin addition, MVA addition mainly influenced the expression of genes related to DNA replication, cell mitosis, pyrimidine metabolism, and certain cell proliferation-promoting signaling pathways (Fig. 2c and S-Fig. 3A, B). Proteomics (S-Table 4), western blot, and RT-PCR results (S-Fig. 3C–F) confirmed that certain genes like dihydrofolate reductase (DHFR) and thymidylate synthetase (TYMS), which are important for DNA synthesis and replication, are upregulated by the addition of MVA.

Wnt signaling controls cell proliferation, migration, and fate specification^26–28^. Wnt signaling is frequently activated in multiple carcinomas, especially in colon cancer^28^. The canonical Wnt signaling is activated by the accumulation of β-catenin^28^, which translocates to the nucleus to act as a coactivator of the TCF/LEF transcriptional factors to initiate gene transcription. Of note, the KEGG enrichment analysis shows that Wnt signaling is regulated by the mevalonate pathway (Fig. 2c). Recent studies have demonstrated that YAP/TAZ mediate alternative Wnt signaling activation^27,28^. Sequencing data shows that the two most significantly upregulated genes by MVA addition are CTGF and CYR61 (Fig. 2b), which are canonical YAP/TAZ target genes^29^. These results suggest that the mevalonate pathway regulates both canonical and alternative Wnt signaling.

**Fig. 4:**
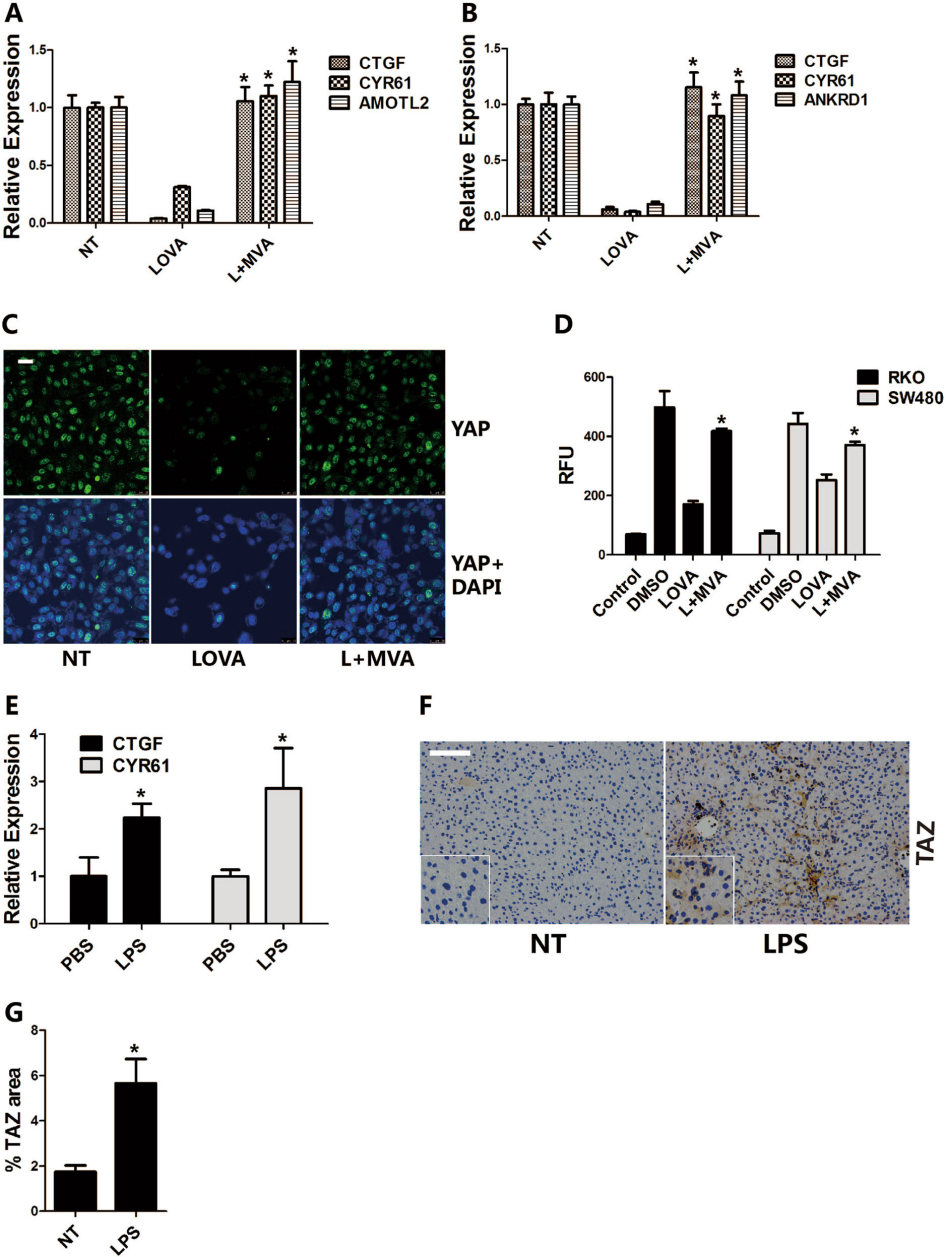
The mevalonate pathway regulates YAP/TAZ mediated alternative Wnt signaling in vitro and in vivo. **a**, **b** Lovastatin treatment suppresses the expression of YAP/TAZ target genes, while exogenous MVA restores them. RKO (**a)** or SW480 (**b)** cells are treated with lovastatin (5 μM) or lovastatin plus MVA (0.5 mM) for 48 h, and RNA is isolated for the RT-PCR assay. **c** Lovastatin treatment decreased the expression of YAP in RKO cells. Cells are treated with lovastatin (5 μM) or lovastatin plus MVA (0.5 mM) for 48 h, then immunofluorescence is performed to detect the level of YAP. **d** Lovastatin treatment suppresses the activity of 8×GTIIC-Lux reporter, while MVA recovered it. 8×GTIIC-Lux reporter is transfected to RKO or SW480 cells, then the cells are treated with lovastatin (5 μM) or lovastatin plus MVA (0.5 mM) for 48 h, and then the luminescence is measured. **e**–**g** LPS treatment upregulated the RNA expression of CTGF and CYR61 (**e**) and the protein level of TAZ (**f**) in mouse liver. Eight to ten weeks male C57/BL6 mice were treated with vehicle (PBS) or LPS (1.5 mg/kg/day) for 7 days, then the liver is harvested for RNA isolation for RT-PCR or fixed for immunohistochemistry. TAZ staining area was quantified (**g**). Data are expressed as means ± SEM. *P < 0.05 (LOVA versus L + MVA; PBS versus LPS)

**Fig. 5:**
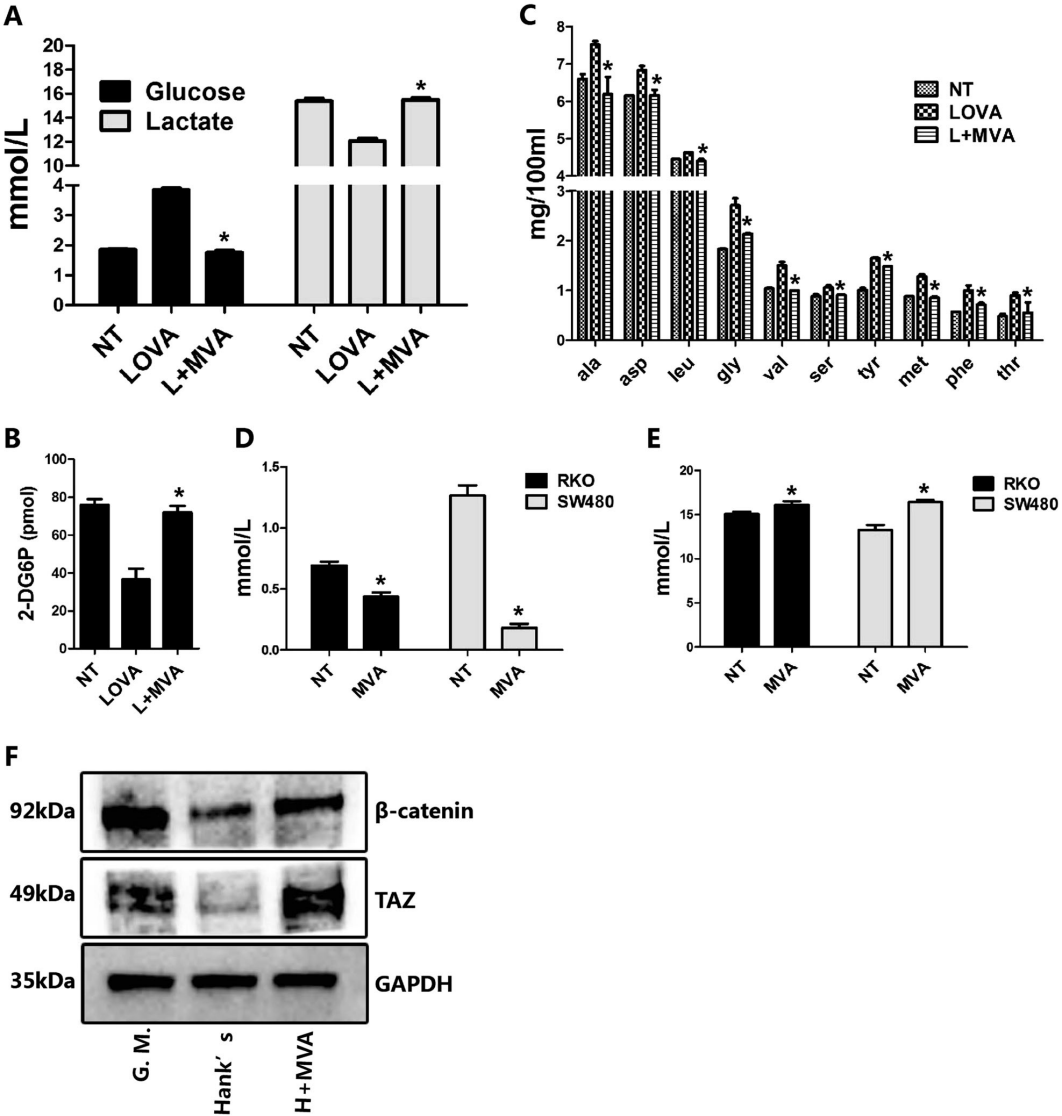
The mevalonate pathway controls nutrient uptake. **a** Lovastatin treatment suppresses glucose uptake and lactate acid production, while exogenous MVA recovered the level of glucose and lactate acid to that of the no-treatment group. RKO cells are treated with lovastatin (5 μM) or lovastatin plus MVA (0.5 mM) for 48 h, then the medium is harvested for glucose and lactate acid determination. **b** Lovastatin treatment suppresses 2-DG uptake in RKO cells, while exogenous MVA recovered it. The cells are treated as described in the Method section. **c** Lovastatin treatment suppresses amino acid uptake, while exogenous MVA recovered the amino acid level to that of the no-treatment group. RKO cells are treated with lovastatin (5 μM) or lovastatin plus MVA (0.5 mM) for 48 h, then the medium is harvested for amino acid determination by mass spectrometry analysis. **d**, **e** MVA itself stimulates glucose uptake and lactate acid production in RKO and SW480 cells. RKO or SW480 cells are cultured in no serum RPMI-1640 medium and supplemented with or without MVA (0.5 mM) for 36 h and the medium is harvested for glucose and lactate acid determination. **f** MVA increases β-catenin and TAZ protein in energy stressed RKO cells. RKO cells are cultured in an energy stress condition (cultured in Hank’s solution, with 5.5 mM glucose) supplemented with or without MVA (0.5 mM) for 12 h and the cells are harvested for western blot assays. Data are expressed as means ± SEM. *P < 0.05 (LOVA versus L + MVA; NT versus MVA)

Next, we performed proteomic analysis and found that lovastatin treatment significantly altered the expression profile of proteins, while MVA addition largely recovered protein expression to the control level (Fig. 2d). Moreover, the proteins upregulated by MVA addition were also significantly relevant to DNA replication and nucleotide excision repair, processes that are related to cell mitosis (S-Figs. 4 and 5A). Of note, lovastatin treatment significantly suppressed ribosomal proteins, which were significantly rescued by exogenous MVA addition (S-Fig. 5A, B). Given that ribosomal proteins are important for mRNA translation, these data suggest that the mevalonate pathway is essential for protein synthesis, a biological process critical for cell mitosis and proliferation. Taken together, the results at both the transcriptome and proteome level explain why lovastatin treatment significantly suppresses cell growth and proliferation.

### The mevalonate pathway regulates multiple signaling pathways relevant to cell proliferation

RNA sequencing results suggest that Wnt signaling is regulated by the mevalonate pathway. To confirm this, we first determined the expression of Wnt target genes by RT-PCR analysis. Lovastatin treatment significantly downregulated Wnt target genes expression in both RKO cells, in which the Wnt pathway is functioning normally, and SW480 cells, in which the APC gene is mutated causing a constitutively active Wnt pathway^30^ (Fig. 3a, b). Exogenous MVA supplementation recovered the suppressed gene expression caused by lovastatin treatment (Fig. 3a, b).

Glycogen synthase kinase-3β (GSK3β), together with casein kinase Iα (CKIα), phosphorylate β-catenin, which initiates subsequent β-catenin ubiquitination and degradation^30,31^. Phosphorylation of GSK3β at serine 9 inhibits its catalytic activity^31^. The results show that lovastatin treatment suppressed GSK3β phosphorylation at serine 9, which led to increased β-catenin phosphorylation and its degradation in both RKO and SW480 cells (Fig. 3c–e). Once β-catenin translocates to the nucleus, it binds to the TCF/LEF transcriptional factor to initiate transcription^28^. Therefore, we thus tested the influence of lovastatin on the luciferase reporter activity which contains TCF/LEF response elements (Fig. 3f, g). As expected, lovastatin treatment suppressed the activity of TCF/LEF reporter, while MVA recovered it. All the above results indicated that the mevalonate pathway controls the activity of canonical Wnt signaling.

YAP/TAZ is a novel mediator of alternative Wnt signaling^29,30^. RNA sequencing data showed that CTGF, CYR61, ANKRD1, and AMOTL2, which are all verified YAP/TAZ target genes^29^, were strikingly suppressed by lovastatin treatment, but upregulated by MVA supplementation. To further confirm that MVA not only regulates canonical Wnt signaling but also controls the alternative Wnt signaling mediated by the transcription factors YAP/TAZ, we performed RT-PCR and confirmed the sequencing results (Fig. 4a, b). In addition, lovastatin triggered a robust decrease of the TAZ protein, while MVA supplement reversed the changes seen in RKO and SW480 cells (Fig. 3c, d). Immunofluorescence results also demonstrated that lovastatin treatment decreased the nuclear YAP accumulation, while it was recovered by MVA addition (Fig. 4c). Consistent with prior findings, lovastatin strongly suppressed the activity of the 8×GTIIC luciferase reporter (Fig. 4d), which contains multimerized responsive elements of TEAD^30^, the main DNA-binding cofactor of TAZ and YAP. These results demonstrate that the mevalonate pathway controls YAP/TAZ mediated signaling.

A recent study from the Medzhitov group showed that sustained inflammation activates the mevalonate pathway in the liver of the mouse^32^. To explore the relevance of our in vitro findings that the mevalonate pathway regulates YAP/TAZ signaling, we treated the mice with PBS or lipopolysaccharide (LPS) to test whether the activated mevalonate pathway by LPS promotes the activity of YAP/TAZ signaling in vivo. It showed that the RNA expression of CTGF and CYR61 were upregulated in the liver by LPS treatment (Fig. 4e). Furthermore, the immunohistochemistry analysis suggested that chronic LPS administration increased the TAZ protein level in the liver (Fig. 4f, g). The results clearly showed that the activated mevanolate pathway in the liver of the mice activated YAP/TAZ signaling. Taken together, these data reveal that the activation of the mevalonate pathway results in the upregulation of YAP/TAZ signaling in vitro and in vivo.

**Fig. 6:**
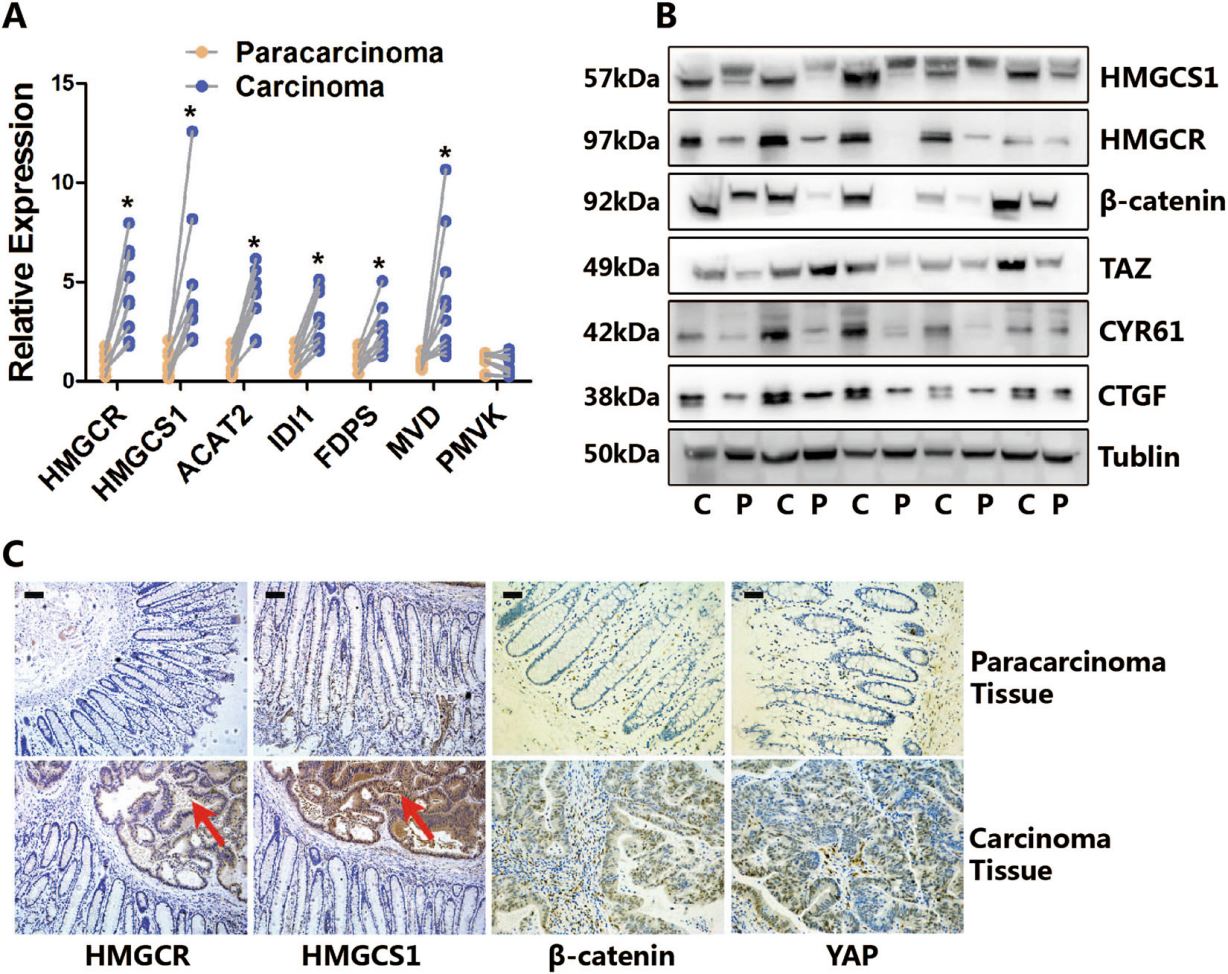
Colon cancer has a higher mevalonate pathway activity than paracarcinoma tissue. **a** Cancer tissue has higher RNA expression of multiple enzymes of the mevalonate pathway than paracarcinoma tissue. RNA expression of the cancer tissue and the paracarcinoma tissue from the same patient are compared. (*n* = 10). **b** Western blot assays of the cancer tissue (C) and the paracarcinoma tissue (P) from the same patient. It shows cancer tissue has higher protein level of HMGCS1, HMGCR, β-catenin, TAZ, CTGF, and CYR61 than paracarcinoma tissue. (*n* = 10). **c** Immunohistochemistry assays of the cancer tissue and the paracarcinoma tissue from the same patient. It shows cancer tissue has higher protein level of HMGCS1, HMGCR, β-catenin, and YAP than paracarcinoma tissue. (*n* = 10). Data are expressed as means ± SEM. *P < 0.05 (Paracarcinoma versus Carcinoma)

mTOR signaling is another pathway that controls cell growth, proliferation, and metabolism^1,6,10^. Here we found that lovastatin treatment also suppressed mTOR signaling, which was reflected by decreased phosphorylation of ribosomal protein S6 kinase beta 1 (S6K1), ribosomal protein S6 (RPS6), and eukaryotic translation initiation factor 4E-binding protein 1 (4E-BP1). As expected, exogenous MVA addition recovered mTOR signaling activity in RKO and SW480 cells (S-Fig. 6A, B), confirming recent findings that mevalonate activates mTOR signaling^33^. Overall, these results indicate that the mevalonate pathway regulates multiple signaling pathways relevant to cell proliferation.

### The mevalonate pathway controls nutrient uptake

Cell growth and proliferation are energy-consuming processes and should be adapted to the exogenous energy supply. However, how cells sense the status of the environmental energy supply is a basic biological issue and has been widely studied^2,3,6,11^. Given that the mevalonate pathway significantly impacts cell growth and proliferation, we investigated whether the mevalonate pathway also controls nutrient uptake. The result shows that lovastatin strongly suppressed glucose uptake and utilization in both RKO and SW480 cells, reflected by a higher concentration of remnant glucose and a lower concentration of lactate in the culture medium (Fig. 5a). Exogenous MVA supplementation completely recovered the level of glucose and lactate to that of the no-treatment group (Fig. 5a). To validate the above result, we next performed a 2-deoxy-d-glucose (2-DG) uptake assay and confirmed that lovastatin suppresses 2-DG uptake, while MVA addition rescues the suppression by lovastatin (Fig. 5b).

Amino acids are another type of nutrient necessary for cell growth and energy supplementation. We thus tested whether the mevalonate pathway also controls the uptake of amino acids. The concentration of multiple amino acids in the medium was higher in lovastatin-treated cells but retune to the level of the no-treatment condition with exogenous MVA addition (Fig. 5c), suggesting that the uptake of amino acids is regulated by the mevalonate pathway.

To further confirm whether MVA itself controls glucose uptake, we designed the following experiment: once the cells reached 80% confluence, the growth medium was changed to basic medium (RPMI-1640, without FBS) which was supplied with or without exogenous MVA. The medium was harvested after 36 h. The results show that MVA addition stimulates glucose uptake and metabolism as seen by the decreased glucose level and increased lactate level in the medium (Fig. 5d, e). This result directly supports the hypothesis that the mevalonate pathway itself is sufficient to control glucose uptake. Although MVA addition stimulates glucose uptake, it does not change the number or the morphology of the cells.

Next, we performed western blot analysis and found that the protein levels of β-catenin and TAZ are lower in an energy stress condition (cultured in Hank’s solution, with 5.5 mM glucose) but can be partly rescued by MVA supplementation (Fig. 5f). This result indicates that the mevalonate pathway is sufficient to activate Wnt and TAZ signaling, which may account for its ability to promote cell growth and proliferation. Together with the findings that deprivation of MVA suppresses cell proliferation, these results indicate that the mevalonate pathway is essential, but not sufficient, for cell proliferation and growth.

### Colon cancer has a higher mevalonate pathway activity than paracarcinoma tissue

The proliferation of cancer cells is much faster than their corresponding normal cells. Thus, we hypothesized that cancer cells may have a higher mevalonate pathway activity to support nutrient uptake and cell proliferation. To test this, we compared the mevalonate pathway activity of colon cancer tissue and the paracarcinoma tissue from the same patient. As expected, the RNA expression of multiple enzymes of the mevalonate pathway was higher in colon cancer tissue than in paracarcinoma tissue (Fig. 6a). Western blot results confirmed that the expression of HMGCR, key enzyme controlling the mevalonate pathway activity, was higher in cancer tissue (Fig. 6b). To further test whether colon cancer tissue has higher activity of the mevalonate pathway, we performed immunohistochemistry (Fig. 6c). These results show that the expression of HMGCS1 and HMGCR in the cancer tissue is much higher than in the paracarcinoma tissue, which is consistent with a recent report by Deng et al.^34^.

As the mevalonate pathway regulates Wnt and YAP/TAZ signaling in vitro, we postulate that the upregulated mevalonate signaling may accompany activated Wnt and YAP/TAZ signaling in carcinoma tissues. Here, we found that the expression of β-catenin, which represents classical Wnt signaling activity, was higher in cancer tissues (Fig. 6b). In addition, the expression of TAZ and its target genes, CTGF and CYR61, was also higher in cancer tissues than in the paracarcinoma tissues from the same patients (Fig. 6b). Taken together, these data suggest that colon cancer tissue has a higher mevalonate pathway activity, as well as increased Wnt and YAP/TAZ signaling.

## Discussion

Cell proliferation and growth should be tightly coordinated with nutrient uptake. However, how the cells perceive the abundance of nutrients in the external environment and then use these nutrients to support cell proliferation is not completely understood. In this study, we explored the role of the mevalonate pathway in the regulation of both cell proliferation and nutrient uptake.

HMG-CoA reductase is the rate-limiting enzyme of the mevalonate pathway, which is also the target of statins. We found that lovastatin significantly suppressed the proliferation of multiple colon cell lines, while the addition of MVA back to the medium completely recovered the proliferation. The results of transcriptome and proteome analysis suggested that the mevalonate pathway significantly regulated multiple signaling pathways related to cell proliferation and growth. In addition, biological processes like cell cycle, DNA replication, mitosis, which are relevant to cell division, are all controlled by the mevalonate pathway. These results explain why the mevalonate pathway controls cell proliferation.

Wnt signaling includes canonical and alternative pathways^29,30^. Canonical Wnt signaling is mediated by β-catenin and the TCF/LEF transcriptional factors^27,28^. It was shown that lovastatin treatment increased the phosphorylation and decreased the protein level of β-catenin. Furthermore, the activity of the TCF/LEF luciferase reporter was significantly suppressed by lovastatin treatment but rescued by MVA addition. Recent studies showed that YAP/TAZ are key mediators of alternative Wnt signaling^29,30^. Here, we found that the YAP/TAZ protein was significantly suppressed by lovastatin treatment and completely rescued by MVA addition, which is consistent with previous findings. Western blot, RT-PCR, and reporter assays further confirmed the role of the mevalonate pathway in the regulation of YAP/TAZ. Of note, mice treated with LPS, which leads to an accumulation of metabolites from the mevalonate pathway in the liver, show increased protein level of YAP/TAZ and their target genes in the liver. These data build on prior reports^32,35^ and confirm that the mevalonate pathway controls YAP/TAZ in vitro and in vivo.

Cell growth and proliferation are energy-consuming processes and should be supported by nutrient uptake. In this study, we found that the mevalonate pathway not only supports cell proliferation but also promotes glucose and amino acid uptake. More importantly, our results indicate that MVA is sufficient to promote glucose uptake, although the mechanism remains to be further explored. Recent studies showed that mevalonate supplementation activates the IGF1 receptor^33^. Given that the IGF1 receptor regulates glucose uptake, this may be a likely mechanism for the effect. Together, these data suggest that the mevalonate pathway is not just for the synthesis of cholesterol but also a key pathway for promoting cell proliferation and energy uptake.

Cancer cells grow and proliferate faster than normal cells, and accordingly, they need more energy to support cell proliferation. Thus, we hypothesized that colon cancer tissue may have a higher mevalonate pathway activity than its paracarcinoma tissue. Indeed, we found that cancer tissues have higher expression of multiple enzymes in the mevalonate pathway. Consistent with the increased expression of the enzymes that catalyze MVA synthesis, Wnt signaling was activated, reflected by the increased protein levels of β-catenin and YAP/TAZ.

**Fig. 7:**
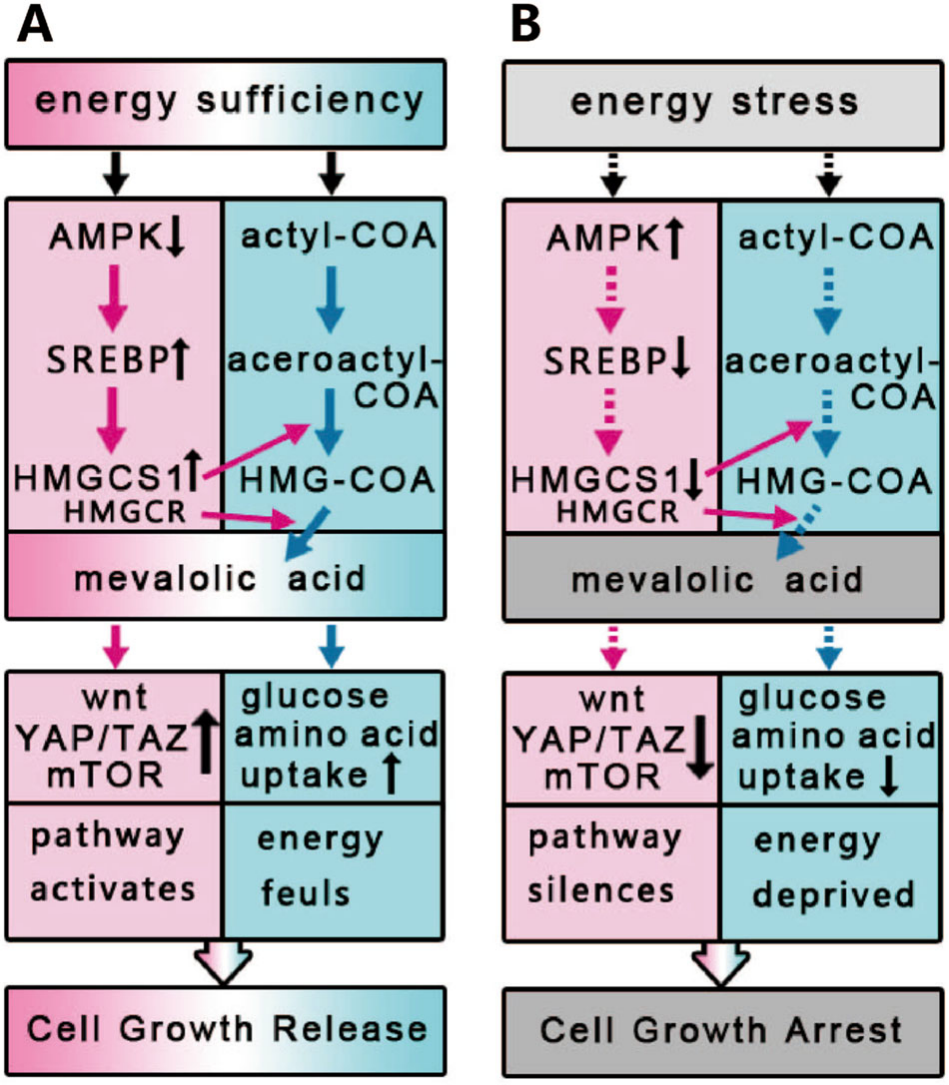
**Model depicting that the mevalonate coordinates energy input and cell proliferation**

AMPK is activated by an increased ratio of AMP/ATP and functions as a key cellular energy sensor^2,3^. It was reported that AMPK can phosphorylate and suppress the activity of sterol regulatory element binding proteins (SREBP-1c and -2)^36^, the transcriptional factors that control gene expression of enzymes in the mevalonate pathway. Except to suppress the activation of SREBPs, AMPK was shown to directly regulate the phosphorylation of HMG-CoA reductase and reduce its enzymatic activity^37^. Based on these and the findings in our study, we established a model depicting how the mevalonate pathway coordinates cell nutrient uptake and proliferation (Fig. 7). When energy is abundant, the expression of enzymes in the mevalonate pathway is normal; in addition, acetyl-CoA, the precursor for synthesis of both ATP and HMG-CoA, is sufficient to support the synthesis of MVA. Both inputs fuel the mevalonate pathway and further activate proliferation-promoting signaling and the uptake of nutrients, simultaneously, leading to cell growth. On the contrary, under conditions of energy stress, the mevalonate pathway is suppressed due to the decreased expression of enzymes and the deficiency of acetyl-CoA for the synthesis of MVA. The silent mevalonate pathway leads to a decrease in nutrient uptake and cell growth arrest.

Given that MVA originates from acetyl-CoA and activates multiple signaling pathways fueling cell proliferation, we conclude that the mevalonate pathway plays a critical role in the coordination of energy input and cell proliferation.

### Reagents and method

#### Cell culture and reagents

HCT116 was cultured in RPMI-1640 supplemented with 10% fetal bovine serum (FBS) and penicillin/streptomycin; RKO, SW480, HT29, CACO2 were cultured in DMEM supplemented with 10% FBS and penicillin/streptomycin. Cells were maintained at 37 °C in the presence of 5% CO_2_ in air. DL-mevalonic acid 5-phosphate (79849), geranylgeranyl pyrophosphate (G6025), farnesyl pyrophosphate (F6892) were purchased from Sigma Aldrich. Lovastatin was purchased from MedChemExpress (Shanghai, China).

#### Western blot and antibodies

Western blot analyses were performed using standard protocols. The primary antibodies used in this study are listed: TAZ (4883S), YAP (P46937), p-YAP (13008T), phospho-GSK-3β (5558), phospho-β-catenin (2009) antibody, and the mTOR pathway antibody kit (9862) were from Cell Signaling Technology; CTGF antibody (PA5–32193) was from Thermofisher Scientific; CYR61, HMGCS1, and GAPDH antibodies were from Sangon Biotech (Shanghai, China); Tublin antibody was from Boster (BM1623, Wuhan, China). HMGCR (ab174830), β-catenin (ab32572), ENC1 (ab124902), TYMS (ab108995), DHFR (ab133546), RhoA (ab187027), RhoB (D160056) antibodies were purchased from Abcam. Western blots were developed using the Immobilon ECL Ultra Western HRP Substrate (Millipore), and images were captured using a ChemiDoc MP Imaging System (Bio-Rad).

#### RNA isolation, reverse transcription and real-time qPCR

Trizol (Life Technologies, CA) was used for total RNA isolation. Following isolation, 500 ng total RNA was reverse transcribed with the All-in-One First-Strand cDNA Synthesis Kit (Genecopoeia, MD) and qPCR was performed using All-in-One qPCR Mix (Genecopoeia, MD) and Applied Biosystems 7500 Real-Time PCR detection system. The gene expression was referred to 36B4. The primer sequences for qPCR assay were as follows:

Human:

HMGCR For: GGACCCCTTTGCTTAGATGAAA; Rev: CCACCAAGACCTATTGCTCTG;

HMGCS1 For: GGGCAGGGCATTATTAGGCTAT; Rev: TTAGGTTGTCAGCCTCTATGTTGAA;

ACAT2 For: GCGGCGCGGACCAT; Rev: CCTGGACAGGAACAGCAGCTA;

IDI1 For: TTTCCAGGTTGTTTTACGAATACG; Rev: TCCTCAAGCTCGGCTGGAT;

FDPS For: CTTCCTATAGCTGCAGCCATGTAC; Rev: GCATTGGCGTGCTCCTTCT;

MVD For: TGAACTCCGCGTGCTCATC; Rev: CGGTACTGCCTGTCAGCTTCT;

PMVK For: CCGCGTGTCTCACCCTTT; Rev: GACCGTGCCCTCAGCTCAT;

AXIN2 For: GCTGACGGATGATTCCATGT; Rev: ACTGCCCACACGATAAGGAG;

ENC1 For: CATTTGTCAGCACCTGGAAA; Rev: TCTCATCGAGTGATGGAGTGA;

BIRC5 For: AGAACTGGCCCTTCTTGGA; Rev: CAAGTCTGGCTCGTTCTCAGT;

BCL2L1 For: CCCAGGGACAGCATATCAG; Rev: AGCGGTTGAAGCGTTCCT;

ANKRD1 For: CGAGATAAGTTGCTCAGCACAG; Rev: GTTCAGTCTCACCGCATCATG;

CTGF For: AGCTGACCTGGAAGAGAACATT; Rev: GCTCGGTATGTCTTCATGCTG;

CYR61 For: AAGAAACCCGGATTTGTGAG; Rev: GCTGCATTTCTTGCCCTTT;

PCNA For: CCTGCTGGGATATTAGCTCCA; Rev: CAGCGGTAGGTGTCGAAGC;

36B4 For: CAACCCAGCTCTGGAGAAAC; Rev: GTGAGGTCCTCCTTGGTGAA.

Mouse:

CTGF For: CTGCAGACTGGAGAAGCAGA; Rev: GCTTGGCGATTTTAGGTGTC;

CYR61 For: CAACCAGTGTACAGCAGCCTA; Rev: GCAGTATTTGGGCCGGTAT;

36B4 For: CAACCCAGCTCTGGAGAAAC; Rev: GAGGTCCTCCTTGGTGAACA.

#### Luciferase assays

8×GTIIC-luciferase reporter (a gift from Stefano Piccolo, Addgene plasmid #34615) and TCF/LEF luciferase reporter (E461A, Promega) assays basically followed the protocol recommended by Promega (Promega, Part# 9PIE461) and our previous publications^27^. RKO and SW480 were used for luciferase reporter transfection. Eighteen hours after plasmids transfection (0.15 μg/cm2), cells were proceeded to ANG II treatment. Twenty-four hours after ANG II (5 μM) addition, the cells were lysed and then the ONE-Glo™ Luciferase Assay System detection reagent (E6110, Promega) was used to measure the luminescence.

#### 2-DG uptake assays

The Glucose Uptake Colorimetric Assay Kit (Biovision, K676) was used to evaluate the 2-DG uptake by RKO cells. Briefly, RKO cells were treated with lovastatin (5 μM) or lovastatin plus MVA (0.5 mM) for 24 h, and the medium was changed to no serum medium (with lovastatin or lovastatin plus MVA) for another 12 h, and then the 2-DG uptake assays were performed according to the manufacturer's instruction.

#### RNA sequencing

RNA sequencing was performed as described in our previous study^27^. Briefly, total RNA was isolated from the MEF using Trizol reagent (Invitrogen). Before library construction, RNA degradation and potential contamination were tested by agarose gel electrophoresis, and RNA integrity and quantitation were checked by Agilent 2100 bioanalyzer. Three micrograms of total RNA was sequenced by Hiseq 2500.

#### Proteomics assay

1. SILAC labeling

The proteomics assay and analysis were done in the PTM biolabs (Hangzhou, China). RKO cells were labeled with either “heavy isotopic amino acids” (l-13C6-lysine/l-13C615N4-arginine) or “light isotopic amino acids” (l-lysine/l-arginine) using a SILAC Protein Quantitation Kit (Pierce, Thermo) according to manufacturer’s instructions. The cells were grown for more than six generations before being harvested, to achieve more than 97% labeling efficiency. After that, the differently labeled cells were treated with lovastatin or lovastatin plus MVA for 40 h, then the cells were washed two times with cold PBS and proceeded to protein extraction.

2. Protein extraction

Samples were sonicated on ice using a high-intensity ultrasonic processor (Scientz) in lysis buffer (8 M urea, 1% Protease Inhibitor Cocktail). The remaining debris was removed by centrifugation at 12,000 *g* at 4 °C for 10 min. Finally, the supernatant was collected and the protein concentration was determined with the BCA kit according to the manufacturer’s instructions.

3. Trypsin digestion

For digestion, the protein solution was reduced with 5 mM dithiothreitol for 30 min at 56 °C and alkylated with 11 mM iodoacetamide for 15 min at room temperature in darkness. The protein sample was then diluted by adding 100 mM NH_4_HCO_3_ to urea concentration less than 2 M. Finally, trypsin was added at 1:50 trypsin-to-protein mass ratio for the first digestion overnight and 1:100 trypsin-to-protein mass ratio for a second 4-h digestion.

4. HPLC fractionation

The tryptic peptides were fractionated into fractions by high pH reverse-phase HPLC using Agilent 300Extend C18 column (5 μm particles, 4.6 mm ID, 250 mm length). Briefly, peptides were first separated with a gradient of 8–32% acetonitrile (pH 9.0) over 60 min into 60 fractions. Then, the peptides were combined into 18 fractions and dried by vacuum centrifuging.

5. LC-MS/MS analysis

The tryptic peptides were dissolved in 0.1% formic acid (solvent A), directly loaded onto a reversed-phase analytical column (15 cm length, 75 μm i.d.). The gradient was comprised of an increase from 6 to 23% solvent B (0.1% formic acid in 98% acetonitrile) over 26 min, 23–35% in 8 min and climbing to 80% in 3 min then holding at 80% for the last 3 min, all at a constant flow rate of 400 nL/min on an EASY-nLC 1000 UPLC system. The peptides were subjected to NSI source followed by tandem mass spectrometry (MS/MS) in Q ExactiveTM Plus (Thermo) coupled online to the UPLC. The electrospray voltage applied was 2.0 kV. The m/z scan range was 350–1800 for full scan, and intact peptides were detected in the Orbitrap at a resolution of 70,000. Peptides were then selected for MS/MS using NCE setting as 28 and the fragments were detected in the Orbitrap at a resolution of 17,500. A data-dependent procedure that alternated between one MS scan followed by 20 MS/MS scans with 15.0 s dynamic exclusion. Automatic gain control was set at 5E4.

6. Database search

The resulting MS/MS data were processed using Maxquant search engine (v.1.5.2.8)^38^. Tandem mass spectra were searched against Swiss-Prot Human database concatenated with reverse decoy database. Trypsin/P was specified as cleavage enzyme allowing up to two missing cleavages. The mass tolerance for precursor ions was set as 20 ppm in the first search and 5 ppm in the main search, and the mass tolerance for fragment ions was set as 0.02 Da. Carbamidomethyl on Cys was specified as fixed modification and oxidation on Met was specified as variable modifications. FDR was adjusted to <1% and minimum score for peptides was set >40.

7. Calculation of differential proteins

The quantification was mean-normalized at peptide level to center the distribution of quantitative values. Protein quantitation was then calculated as the median ratio of corresponding unique or razor peptides for a given protein. Two-sample, two-sided *t*-tests were used to compare expression of proteins. In general, a significance level of 0.05 was used for statistical testing, and we reported the *P* value or significance level any time a statistical test was performed.

### Statistical analysis

Comparisons between two groups were assessed by the unpaired Student’s *t*-test (2-tail) while multi group analysis was assessed by the one-way analysis of variance (ANOVA) test (2-tail). The data are represented as mean ± standard error of mean (SEM) and the significance level was set at 0.05 (two-sided). Differential expression analysis of two conditions/groups (two biological replicates per condition) was performed using the DESeq R package (1.10.1). The resulting *P*-values were adjusted using the Benjamini and Hochberg’s approach for controlling the false discovery rate. Genes with an adjusted *P* < 0.05 found by DESeq and Log_2_ fold change is greater than ±1 were assigned as differentially expressed.

## Supplementary information

S-Fig 1

S-Fig 2

S-Fig 3

S-Fig 4

S-Fig 5

S-Fig 6

S-Table 1

S-Table 2

S-Table 3

S-Table 4

supplementary figure legends
