# Supplementary figures and images for: The mevalonate coordinates energy input and cell proliferation
Source: Cell Death Dis. 2019 Apr 11;10(4):327. doi: 10.1038/s41419-019-1544-y (PMC6459916; doi:10.1038/s41419-019-1544-y)

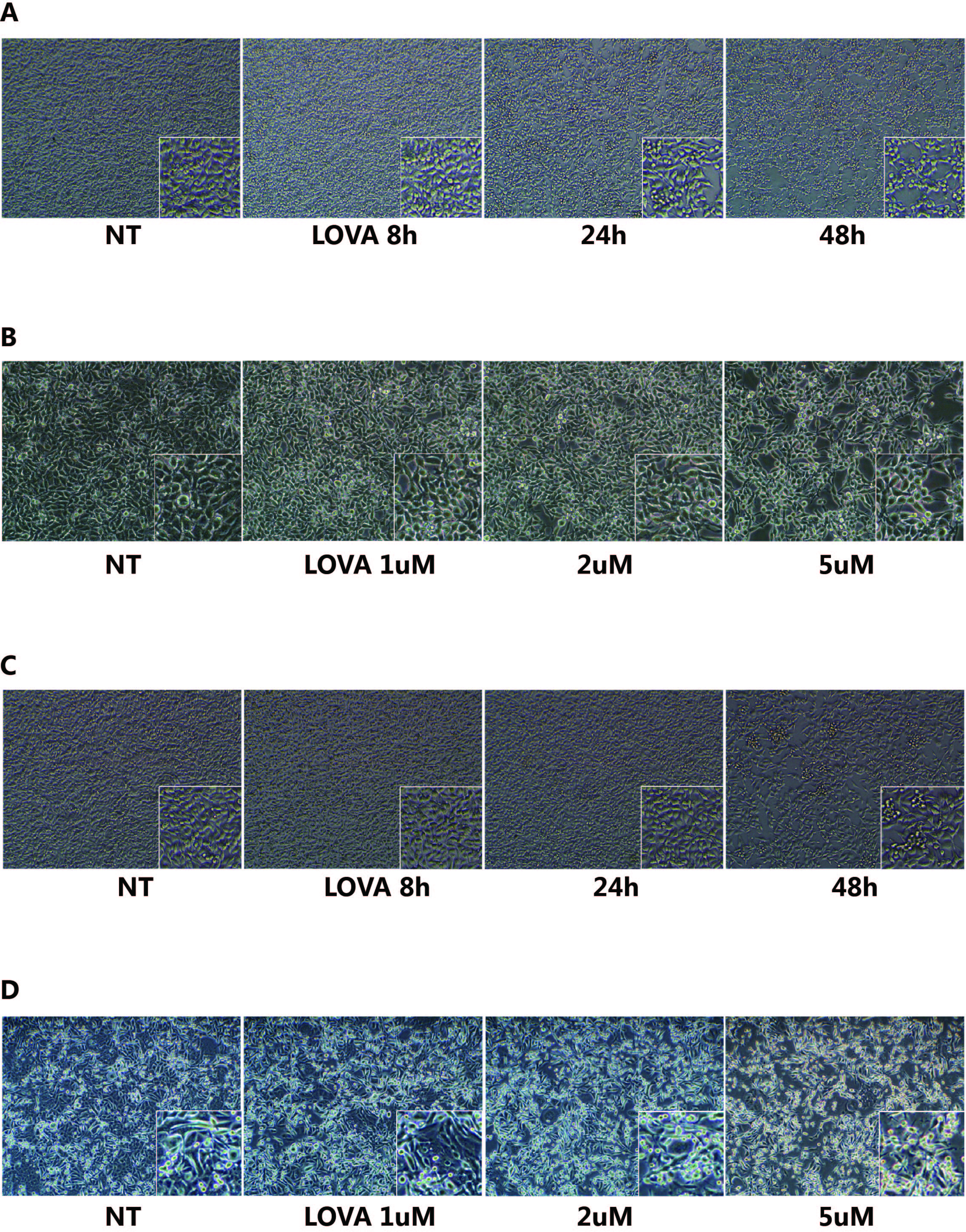

Supplement: Supplementary file 1 — S-Fig 1 [file 41419_2019_1544_MOESM1_ESM.jpg]

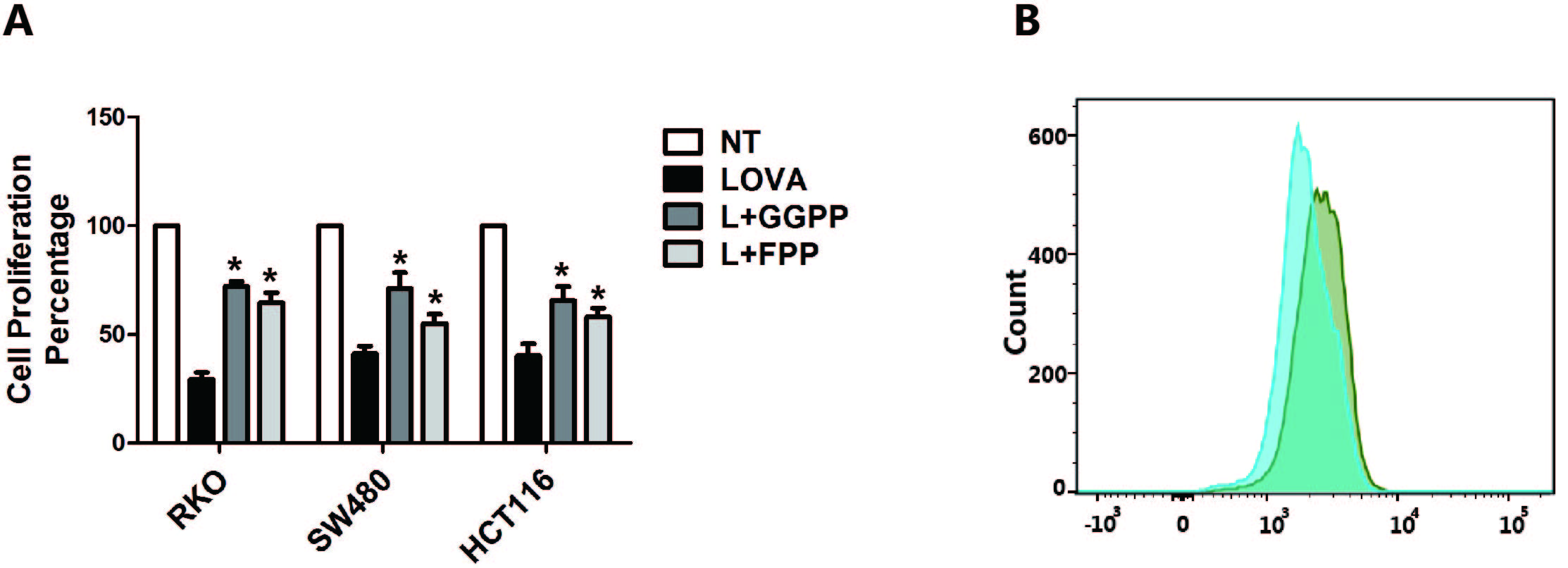

Supplement: Supplementary file 2 — S-Fig 2 [file 41419_2019_1544_MOESM2_ESM.jpg]

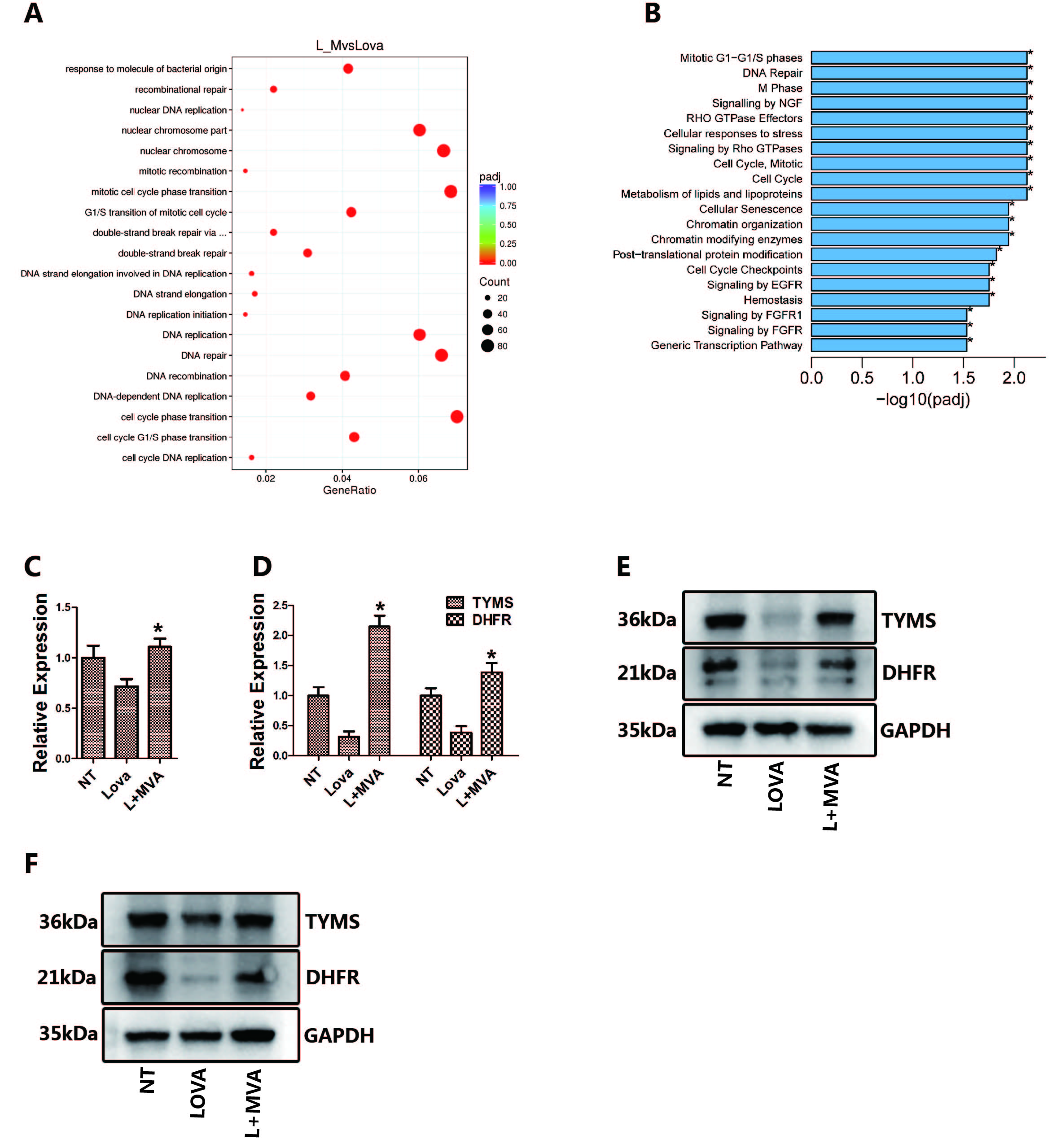

Supplement: Supplementary file 3 — S-Fig 3 [file 41419_2019_1544_MOESM3_ESM.jpg]

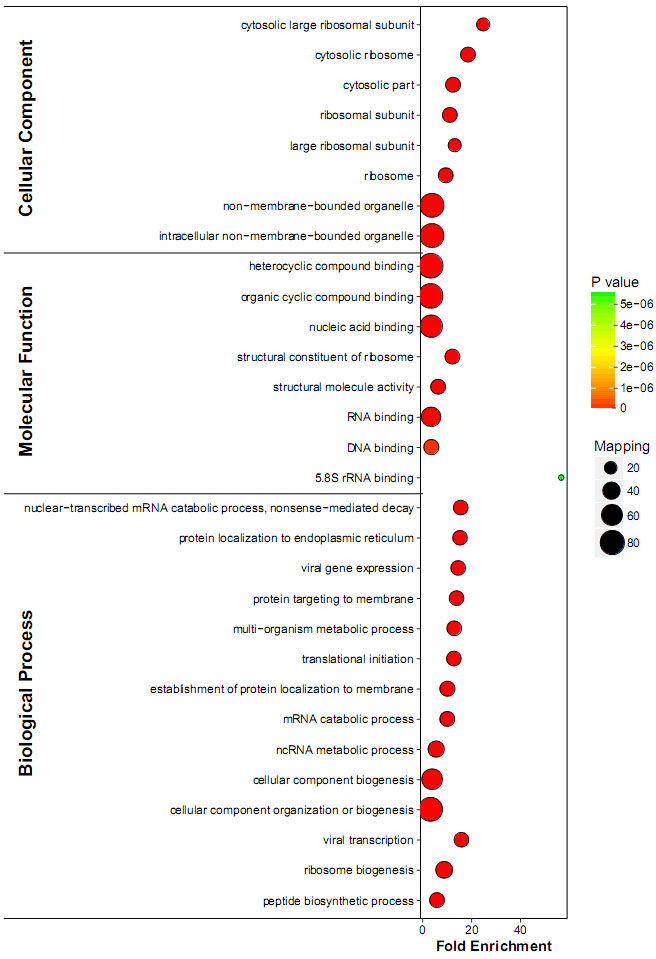

Supplement: Supplementary file 4 — S-Fig 4 [file 41419_2019_1544_MOESM4_ESM.jpg]

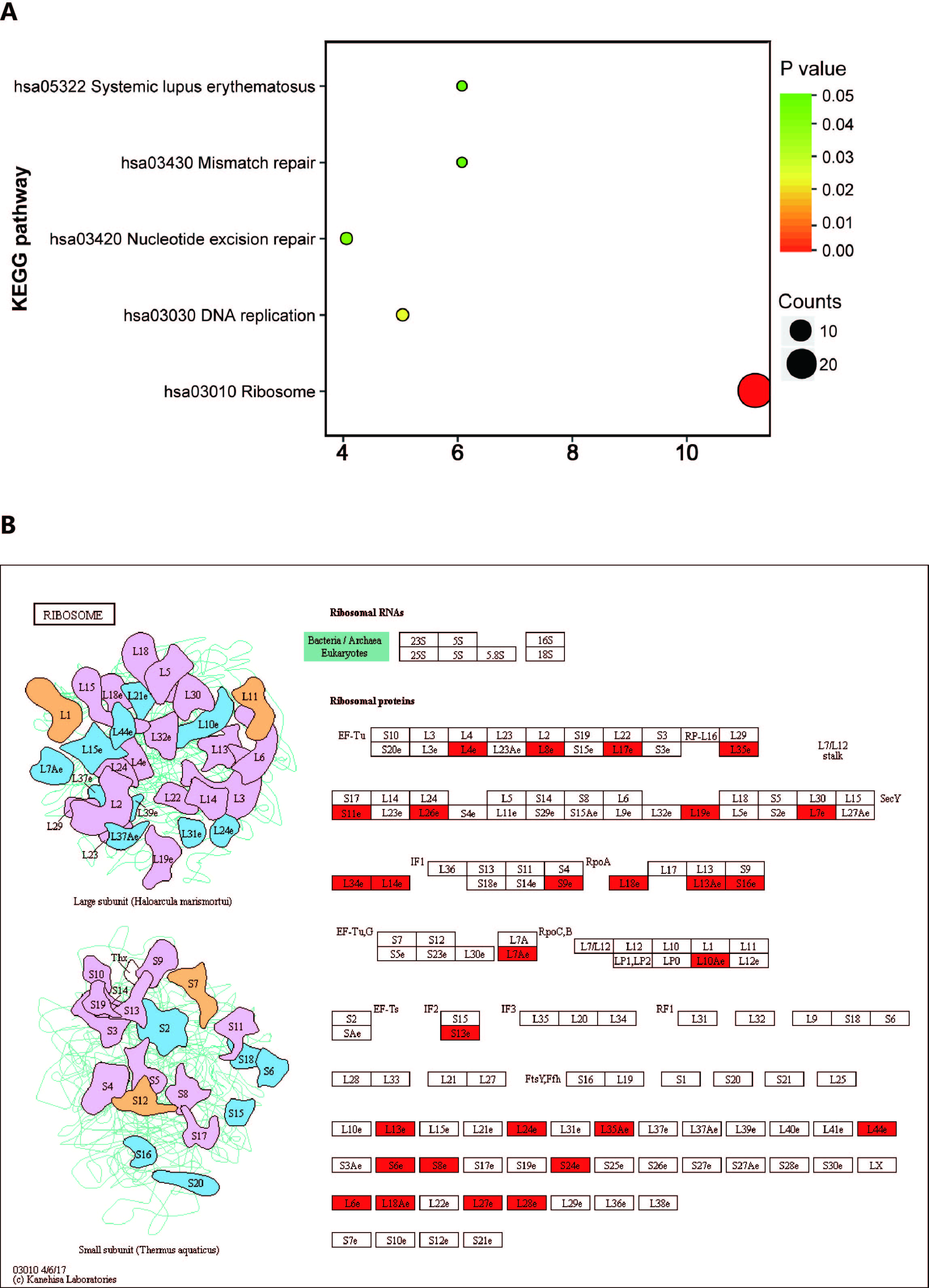

Supplement: Supplementary file 5 — S-Fig 5 [file 41419_2019_1544_MOESM5_ESM.jpg]

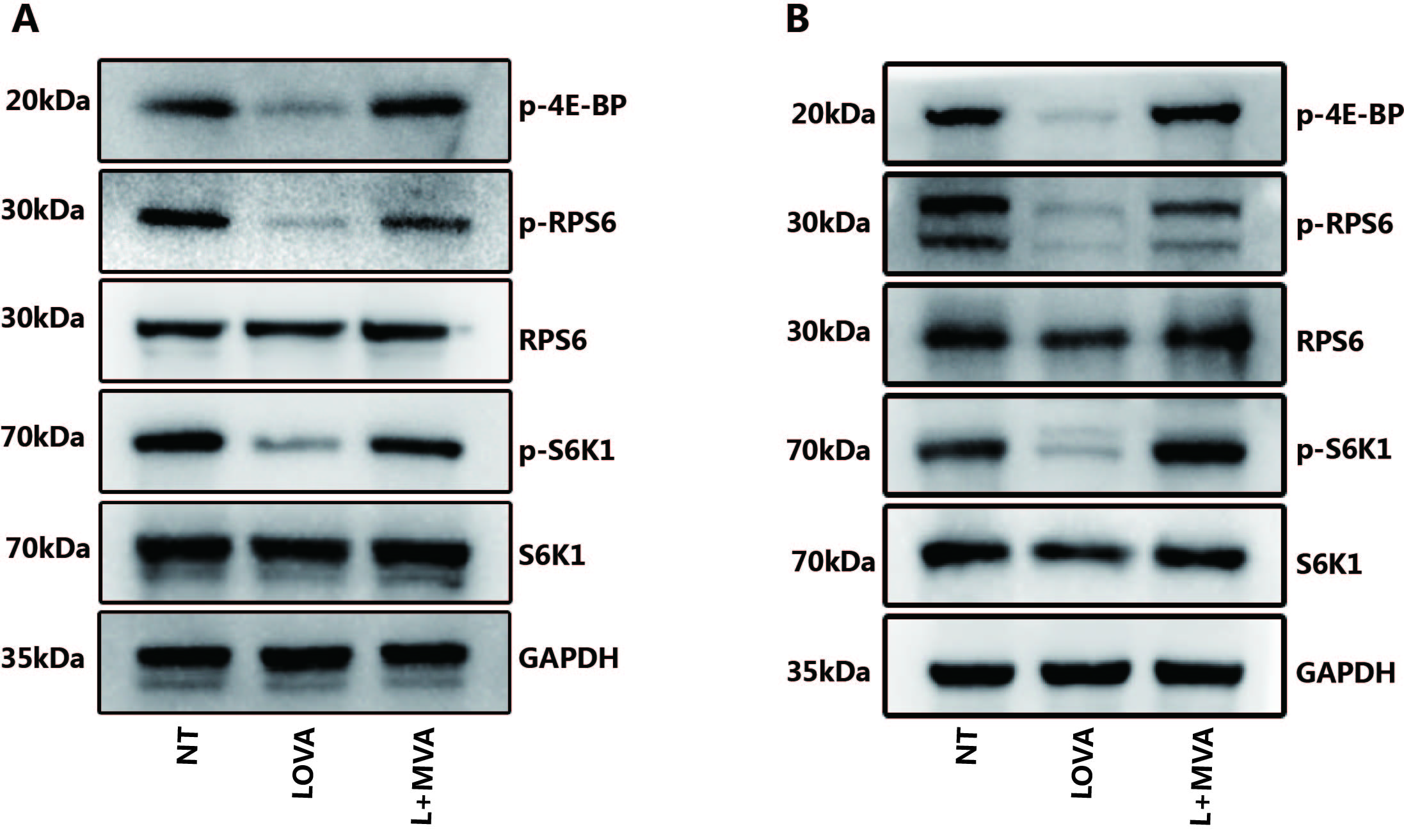

Supplement: Supplementary file 6 — S-Fig 6 [file 41419_2019_1544_MOESM6_ESM.jpg]
