# Supplementary material for: The mevalonate coordinates energy input and cell proliferation
Source: Cell Death Dis. 2019 Apr 11;10(4):327. doi: 10.1038/s41419-019-1544-y (PMC6459916; doi:10.1038/s41419-019-1544-y)
Supplement: Supplementary file 11 — supplementary figure legends [file 41419_2019_1544_MOESM11_ESM.doc]

**Supplemental figures legends:**

**S-Fig. 1**

A-D. RKO or SW480 cells were treated with lovastatin at different time-points and concentrations (RKO: A, B; SW480: C, D).

**S-Fig. 2**

A. Lovastatin treatment suppresses the proliferation of colon cancer cells, but exogenous GGPP or FPP partially restores the cell proliferation. The cells are treated with lovastatin (5μM) or lovastatin plus GGPP or FPP (both at 20μM) for 48 hours, and then the ATP level is measured to evaluate cell proliferation.

B. Lovastatin treatment downregulates the expression of PCNA protein detected by FACS assay. RKO cells are treated with lovastatin for 48 hours, then the cells are proceeded to FACS assay (Green: NT; Blue: lovastatin).

**S-Fig. 3**

A, B. GO (A) and Rectome (B) enrichment of the differentially expressed genes of the RKO cells (lovastatin plus MVA vs lovastatin). RKO cells are treated with lovastatin (5μM) or lovastatin plus MVA (0.5mM) for 48 hours and total RNA is isolated for RNA sequencing.

C, D. Lovastatin treatment suppresses RNA expression of TYMS in SW480 cells (C), and TYMS and DHFR in RKO cells (D), while exogenous MVA addition recovers their expression. RKO or SW480 cells are treated with lovastatin or lovastatin plus MVA for 48 hours, then the RNA is isolated for RT-PCR assays.

E, F. Lovastatin treatment suppresses the protein expression of TYMS and DHFR in RKO (E) and HCT-116 cells (F), while exogenous MVA addition recovers their expression. RKO or SW480 cells are treated with lovastatin or lovastatin plus MVA for 48 hours, then the protein is isolated for western blot assays.

Data are expressed as means ± SEM. *P < 0.05.

**S-Fig. 4**

GO enrichment of the differentially expressed proteins of the RKO cells (lovastatin plus MVA vs lovastatin). RKO cells were labeled as described in Method section. Once labeled with isotopic amino acids, RKO cells are treated with lovastatin (5μM) or lovastatin plus MVA (0.5mM) for 48 hours and then proceeded for proteomic assay.

**S-Fig. 5**

A. KEGG enrichment of the differentially expressed proteins of the RKO cells (lovastatin plus MVA vs lovastatin).

B. Ribosomal proteins are significantly regulated by the mevalonate pathway. The red marked are the upregulated ribosomal proteins by MVA supplementation.

**S-Fig. 6**

A, B. Western blot assays of the mTOR signaling components of RKO (A) and SW480 (B) cells treated with lovastatin or lovastatin plus MVA. RKO or SW480 cells are treated with lovastatin (5μM) or lovastatin plus MVA (0.5mM) for 48 hours, then the protein is isolated for western blot assay.

**S-Tab. 1, 2, 3**

Differentially expressed genes detected by RNA sequencing assay in RKO cells treated with lovastatin (5μM) or lovastatin plus MVA (0.5mM).

S-Tab. 1: lovastatin vs NT;

S-Tab. 2: lovastatin+MVA vs lovastatin;

S-Tab. 3: lovastatin+MVA vs NT.

**S-Tab. 4**

Differentially expressed proteins detected by proteomic assay in RKO cells treated with lovastatin (5μM) or lovastatin plus MVA (0.5mM).
